# Hepatic Coccidiosis in Wild Rabbits in Greece: Parasite Detection on Liver Imprints and the Associated Biochemical Profile

**DOI:** 10.3390/vetsci10040248

**Published:** 2023-03-26

**Authors:** Labrini V. Athanasiou, Constantina N. Tsokana, Dimitrios Doukas, Maria C. Kantere, Panagiotis D. Katsoulos, Georgios I. Papakonstantinou, Eleni G. Katsogiannou, Anna Dedousi

**Affiliations:** 1 Clinic of Medicine, Faculty of Veterinary Medicine, School of Health Sciences, University of Thessaly, 43100 Karditsa, Greece; 2 Laboratory of Pathology, Faculty of Veterinary Medicine, School of Health Sciences, University of Thessaly, 43100 Karditsa, Greece; 3 Clinic of Farm Animals, Faculty of Veterinary Medicine, Aristotle University of Thessaloniki, 54627 Thessaloniki, Greece; 4 Veterinary Research Institute, Hellenic Agricultural Organization DIMITRA, 57001 Thessaloniki, Greece

**Keywords:** *Eimeria stiedae*, Greece, hepatic coccidiosis, liver biochemical variables, liver imprints, wild rabbits

## Abstract

**Simple Summary:**

Hepatic coccidiosis is a severe rabbit parasitic disease caused by *Eimeria stiedae.* It may be fatal for rabbits and leads to important economic losses. The disease is well described in domestic rabbits, but little is known about infection with *E. stiedae* in wild rabbits. In this study, we investigated (a) whether wild rabbits from the island of Lemnos, Greece, where this species is overpopulated, are infected with *E. stiedae* and (b) what the effects are of this infection on their liver function. Based on our findings, 13.3% of the wild rabbits included in this study were infected with *E. stiedae.* Moreover, we observed several alterations in the liver variables in infected individuals compared to the non-infected ones, which were indicative of the negative effects of *E. stiedae* infection on the liver function of wild rabbits. This study adds to the current knowledge on the pathogens affecting wild rabbits and those circulating in this population on the island of Lemnos, Greece, and shows that *E. stiedae* infection impairs liver function in wild rabbits.

**Abstract:**

(1) Background: Rabbit hepatic coccidiosis, caused by *Eimeria stiedae*, is a devastating disease with high morbidity and mortality rates. The disease is well described in rabbits, but little is known about *E. stiedae* infection in wild rabbits. In this study, we investigated the presence of *E. stiedae* infection in wild rabbits from the island of Lemnos, Greece, where this species is overpopulated, and the effects of infection on common hepatic biomarkers. (2) Methods: We used liver impression smears to detect the coccidian oocysts, and we defined the liver biochemical profile of the infected individuals. (3) Results: Overall, 13.3% of the liver imprints examined were positive for the presence of coccidial oocysts. The activities of liver enzymes, that is, alanine aminotransferase (ALT), aspartate aminotransferase (AST) and glutamyltransferase (GGT), as well as globulins (GLOB), were increased while the concentrations of albumins (ALB), total proteins (TP) and the albumin to globulin (A/G) ratio were decreased in the infected individuals compared to the non-infected ones. (4) Conclusions: This study adds to the current knowledge on the pathogens affecting wild rabbits and those circulating in this population on the island of Lemnos, Greece. Moreover, we showed that *E. stiedae* infection exerts pathological effects on the hepatocyte integrity and liver function of wild rabbits, as reflected by the abnormal values of liver injury and dysfunction biomarkers.

## 1. Introduction

Coccidiosis is a highly contagious parasitic disease caused by monoxenous coccidia of the genus *Eimeria* Schneider, 1875. Eleven coccidian species infected domestic and wild rabbits but only a few of them were implicated in clinical disease. Two types of coccidiosis were described: the intestinal form—caused by the coccidian species classified as non-pathogenic (*Eimeria coecicola*), slightly pathogenic (*E. perforans, E. exigua* and *E. vejdovskyi*), mildly pathogenic or pathogenic (*E. media, E. magna, E. piriformis* and *E. irresidua*), and highly pathogenic (*E. intestinalis* and *E. flavescens*), and the hepatic form caused by *E. stiedae*. The latter species has the highest pathogenicity and causes the most severe form of disease [1,2]. 

Hepatic coccidiosis is a devastating disease with high morbidity and mortality rates that results in important economic losses in rabbit farms [3,4]. Young rabbits, from weaning to three-month-old, are more susceptible to infection; mortality rates can be as high as 80%. The adults serve as carriers and a source of infection through the shedding of oocysts [4]. Group housing of rabbits that belong to different age groups, husbandry practices, and the inadequate control of concurrent infections are important risk factors associated with coccidiosis in domestic rabbits [5]. 

The transmission of *E. stiedae* occurs through the fecal-oral route [2]. Once ingested, the oocysts develop into sporozoites which penetrate the duodenal mucosa and migrate to the mesenteric lymph nodes within 12 h [6]. The parasites travel to the liver, where they colonize the epithelium of bile ducts. There, multiple asexual schizogony cycles, gametogony, and the formation of oocysts take place [7]. The bile duct cells burst, the oocysts are released via the bile into the intestines, and they are passed subsequently to the environment via feces [2].

Experimental studies showed that rabbits developed clinical signs at 20 days post-infection. The clinical manifestations include anorexia, diarrhea, lethargy, rough and dull hair and glazed eyes. The disease results in poor weight gain, liver enlargement, ascites, icterus, distended abdomen and finally, death. On top of the premature loss of rabbits, *E. stiedae* may predispose infected animals to other diseases [4,8,9]. 

The post-mortem findings confirmed that hepatic eimeriosis significantly disturbed the liver function: hepatomegaly, multifocal yellowish nodules diffusely spread over the liver surface and in the parenchyma, and considerably dilated bile ducts, biliary hyperplasia and enlarged gallbladder were commonly reported [9,10,11,12]. The histopathological examination shows the hyperplasia of the lining epithelium of the portal areas in the bile duct and coagulative necrosis of the hepatic cells surrounded by inflammatory cells (eosinophils, lymphocytes and plasma cells) [8,11,13]. 

Liver damage and/or dysfunction were further reflected in the alterations of the blood and biochemical variables, as shown previously in experimental studies. A significant increase in the serum alanine aminotransferase (ALT), aspartate aminotransferase (AST), glutamyltransferase (GGT) and lactate dehydrogenase (LDH) activities were recorded while albumin levels, alkaline phosphatase (ALP) activity and cholesterol levels appeared to decrease [4,8,14,15]. As for the blood variables, the values of haematocrit (HCT), haemoglobin (Hg), and mean corpuscular volume (MCV), the counts of erythrocytes, lymphocytes and platelets, and the fibrinogen concentrations all decreased [4,15]. On the contrary, the leukocyte and eosinophil counts increased [4,8,15], and the prothrombin time (PT), the activated partial thromboplastin time (APTT), and the thrombin time (TT) were extended [8]. 

The routine diagnosis of coccidiosis is based on clinical manifestations and the detection of *E. stiedae* oocysts in faeces using faecal flotation [16]. Oocysts of *E. stiedae* have a characteristic extra membrane compared to other coccidian species, called a veil, which, however, can only be visualized using electron microscopy [17]. Other laboratory methods, such as polymerase chain reaction (PCR) and enzyme-linked immunosorbent assay (ELISA), have been shown to be useful tools for the diagnosis of coccidiosis [18,19,20,21]. Early detection of *E. stiedae* DNA using PCR in liver samples, as early as 12 days post-infection, has been shown previously in an experimental study [20]. The complete blood count and biochemical tests, as well as ultrasonographic examinations, can support the diagnosis, but their findings are not disease-specific [4,8]. In practice, an accurate diagnosis of hepatic coccidiosis was based on the postmortem examination of diseased rabbits, the identification of lesions in the affected organs such as the liver and bile duct, and their confirmation with oocysts detection [3]. 

The impression of smear examinations following necropsy is a traditional method for the diagnosis of hepatic coccidiosis that presents several advantages; it is simple and quick, and usually, the smears are of high cellularity and good diagnostic quality. The liver samples contain high numbers of coccidial organisms in various stages of development, from early gametogonous stages to fully formed oocysts [3,22]. 

Several *Eimeria* spp. have been found to infect wild rabbits [23,24]. In a study conducted in the UK, the authors reported that *E. stiedae* was the causative agent of the majority of white-spotted liver lesions in wild rabbits [9]. Although hepatic coccidiosis has been well described in rabbits—especially under experimental conditions—[4,8], little is known about the occurrence of natural *E. stiedae* infection in wild rabbits and its pathological effects. In this study, we investigated the presence of *E. stiedae* infection in a population of wild rabbits (*Oryctolagus cuniculus*) from the island of Lemnos, Greece, where this species is overpopulated, using liver impression smears to detect the coccidian oocysts. Moreover, we defined the biochemical profile of the infected individuals based on the analysis of liver biomarkers and their alterations in hepatic coccidiosis in wild rabbits. 

## 2. Materials and Methods

### 2.1. Animals 

Hunting harvested wild rabbits were sampled during the hunting season 2019–2020 on the island of Lemnos, which was set by the competent authorities (Hellenic Government Gazette 3137/6-8-2019, issue B). Hunting on this island takes place usually as a population control measure to prevent crop damage in compliance with Greek Legislation (Hellenic Government Gazette 3137/6-8-2019, issue B) [25]. The authors declare that no animals were sacrificed while conducting this study and that all ethical standards were followed according to the relevant national and European regulations on animal use and welfare (Directive 2010/63/EC).

### 2.2. Blood Samples and Biochemical Analyses

For the purpose of a previous study, blood samples were taken from the heart of wild rabbits within 3 h after their death, and sera had been recovered after blood clotting and consequent centrifugation. A total of 60 sera samples were selected from these samples using the following criteria: (a) the absence of hemolysis, (b) an adequate volume for biochemical analyses, and (c) a negative result for the presence of antibodies against each pathogen tested in the previous study namely *Leishmania infantum*, *Toxoplasma gondii*, *Anaplasma phagocytophilum* and *Babesia caballi*. 

The serum total protein (TP) concentration was immediately measured using a temperature-compensated refractometer (Reichert TS Meter refractometer, Model 1310400A, Reichert Scientific Instruments Buffalo, NY, USA) [26]. Albumin (ALB) concentration and aspartate aminotransferase (AST), alanine aminotransferase (ALT), alkaline phosphatase (ALP), and gamma glutamyl transpeptidase (GGT) activities were determined in the serum using an automated biochemical analyzer (Biosystems Analyzer A25). Globulin (GLOB) was calculated as the difference between Total Protein and Albumin, while the Albumin to Globulin (A/G) ratio was calculated using the equation A/G ratio = albumin/(total protein − albumin). 

### 2.3. Liver Imprints 

The liver tissue from each animal was imprinted on a slide, fixed in methanol, and Hemacolor stained. A microscopical examination of stained imprints was used to reveal the presence of coccidia.

### 2.4. Statistics

Data were analyzed using the statistical program JASP 16.1. The normality of data distribution was assessed with the Shapiro–Wilk test; depending on the normality results, the Student t-test or Mann–Whitney test was run to determine the significance of the differences between groups for each variable evaluated. A value of *p* ≤ 0.05 was considered significant in all comparisons. To maximize the statistical information provided, a raincloud plot was created by combining a cloud of points with a box plot and a one-sided violin plot [27]. 

## 3. Results

### 3.1. Liver Imprints

**Figure 1:**
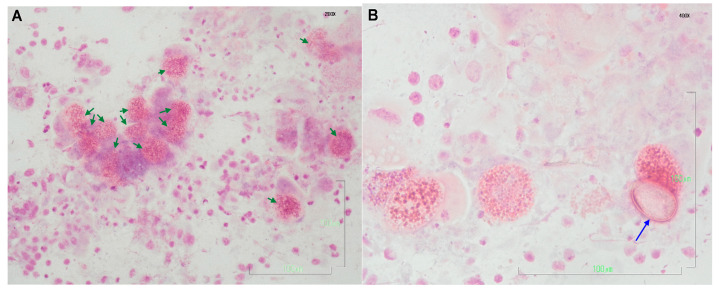
Selected cytological images of liver imprints obtained from wild rabbits. The presence of numerous protozoan structures (in various stages of development) is morphologically compatible with *E. stiedae*; based on the current knowledge, this is the only *Eimeria* sp. parasitizing the rabbit liver. (**A**) Many mature multinucleate microgamonts in the intrahepatic bile duct epithelial cells (green arrows). Hemacolor stain, 200×, bar 100 μm; (**B**) Some multinucleate microgamonts and one ovoid shaped unsporulated oocyst with the characteristic double (composed of two layers) wall (blue arrow). Hemacolor stain, 400×, bar 100 μm.

Coccidia was retrieved in 8 out of the 60 liver imprints (13.3%) (Figure 1) in high numbers, together with hepatobiliary parenchymal cells and a small number of inflammatory cells (Figure 1). The coccidial organisms were in various stages of development as well as fully formed oocysts with an ovoid to ellipsoidal shape. Based on the results of the liver imprint examination, the infected individuals were assigned to Group A, and the non-infected individuals were assigned to Group B.

### 3.2. Biochemical Analyses

The mean values for ALT, AST and GGT activities, the TP, ALB and GLOB concentrations, and the A/G ratio for Group A and Group B are shown in Table 1. The reference intervals that we used in this study for the above-mentioned variables are also presented in Table 1. These are the reference intervals for live domestic rabbits that we used in our laboratory for the specific analyzer used in this study. These reference intervals were used after conducting a method comparison study between this analyzer and an analyzer with established reference intervals (IDEXX VetTest* Chemistry Analyzer) while also using Passing Bablok analysis and Bland–Altman plots and proving that the two methods could be used interchangeably according to the relevant guidelines [28,29,30].

**Figure 2:**
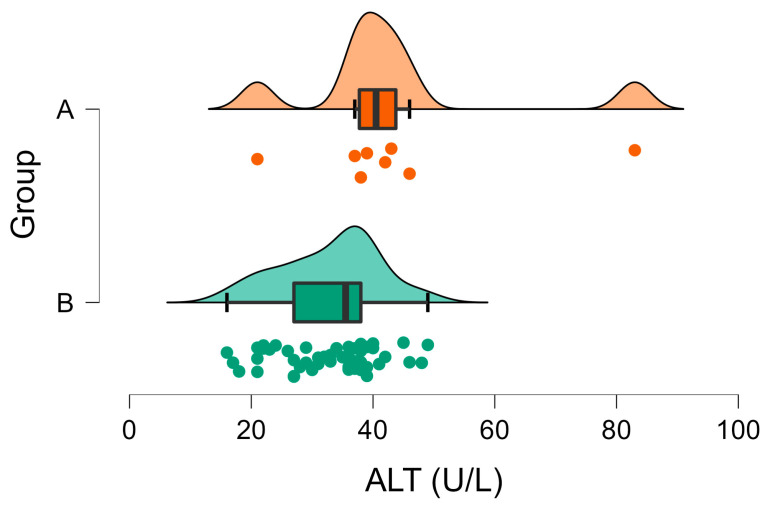
Raincloud plot of ALT activity (U/L) in Group A (wild rabbits with positive *Eimeria* liver imprints) and Group B (wild rabbits with negative *Eimeria* liver imprints). The data distribution (cloud) with jittered raw data (the rain) and the central tendency and error (boxplot) are illustrated.

In Group A, the ALT activities ranged from 21 to 83 U/L and exceeded the upper reference limit for domestic rabbits in only one case (1/8, 12.5%). In Group B, they ranged from 16 to 49 U/L and did not exceed the reference interval in all cases (Figure 2). Moreover, a significant difference between the mean ALT activities of the two groups was observed (Table 1, Figure 2).

**Figure 3:**
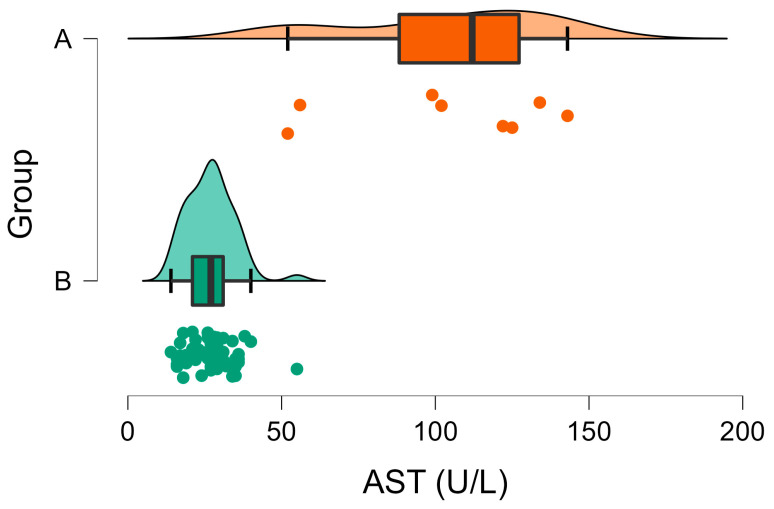
Raincloud plot of AST activities (U/L) in Group A (wild rabbits with positive *Eimeria* liver imprints) and Group B (wild rabbits with negative *Eimeria* liver imprints). The data distribution (cloud) with jittered raw data (the rain) and the central tendency and error (boxplot) are illustrated.

Regarding the AST activities in Group A, they ranged widely from 52 to 143 U/L and exceeded the reference values in 75% (6/8) of the wild rabbits. On the contrary, in Group B, none of the animals had AST activities above the upper reference limit (range 14–55 U/L) (Figure 3). A significant difference was observed between the mean AST activities in the two groups (Table 1, Figure 3).

**Figure 4:**
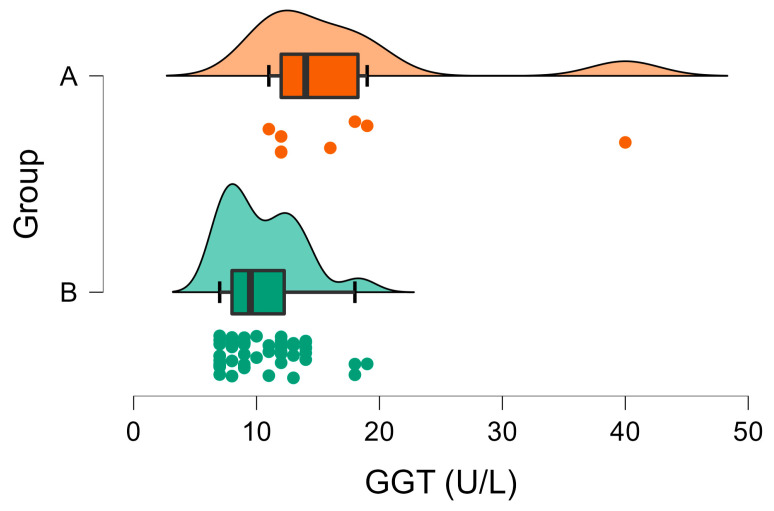
Raincloud plot of GGT activities (U/L) in Group A (wild rabbits with positive *Eimeria* liver imprints) and Group B (wild rabbits with negative *Eimeria* liver imprints). The data distribution (cloud) with jittered raw data (the rain) and the central tendency and error (boxplot) are illustrated.

The GGT activities were above the reference values in 100% (8/8) of the animals in Group A, ranging from 11 to 40 U/L. Similarly, the animals in Group B had GGT activities ranging from 7 to 19 U/L, which also exceeded the reference values but presented a much narrower distribution, as it is depicted in Figure 4. The mean GGT activity in Group A was statistically higher than the corresponding value in Group B (Table 1, Figure 4).

**Figure 5:**
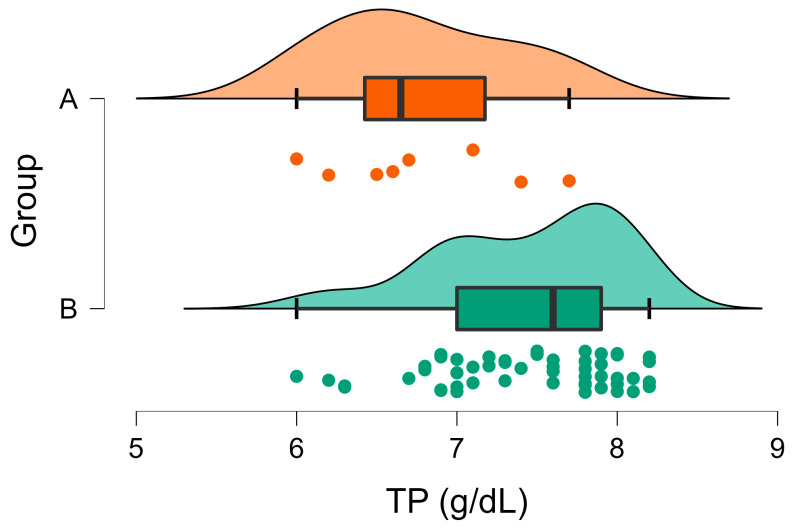
Raincloud plot of TP concentrations (U/l) in Group A (wild rabbits with positive *Eimeria* liver imprints) and Group B (wild rabbits with negative *Eimeria* liver imprints). The data distribution (cloud) with jittered raw data (the rain) and the central tendency and error (boxplot) are illustrated.

As for TP concentrations, they were within the reference intervals for all the animals in Group A except for the two cases with higher values (range 6.0–7.7 g/dL). In Group B, none of the animals had TP concentrations below the reference values, but 67.3% had values greater than 7.2 g/dL (the upper limit of the reference interval) (Figure 5). Moreover, a significant difference was observed between the mean TP in the two groups (Table 1, Figure 5).

**Figure 6:**
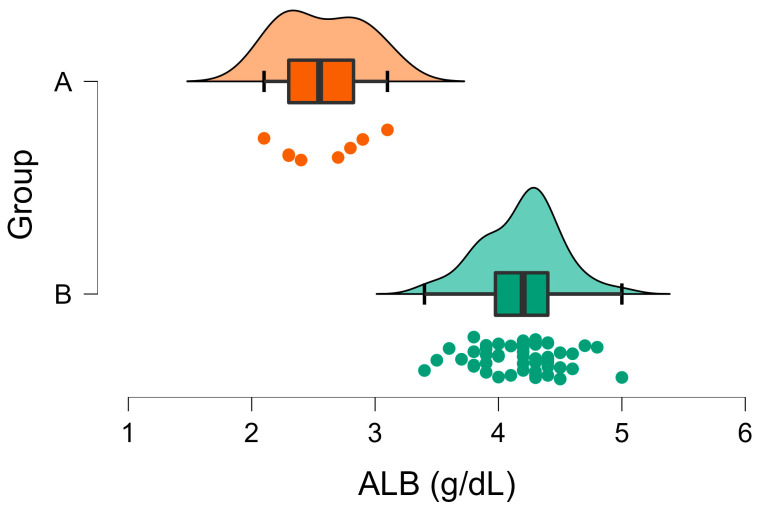
Raincloud plot of ALB concentrations (U/l) in Group A (wild rabbits with positive *Eimeria* liver imprints) and Group B (wild rabbits with negative *Eimeria* liver imprints). The data distribution (cloud) with jittered raw data (the rain) and the central tendency and error (boxplot) are illustrated.

The albumin concentrations were below the lower reference limit in 50% of the animals in Group A, ranging from 2.1 to 3.1 g/dL. In Group B, 98% of the individuals had ALB values within the reference interval (3.4–5.0 g/dL), with only one case (1/52) experiencing an elevated ALB concentration (Figure 6). The mean ALB concentration was significantly lower in group A compared to Group B (Table 1, Figure 6).

**Figure 7:**
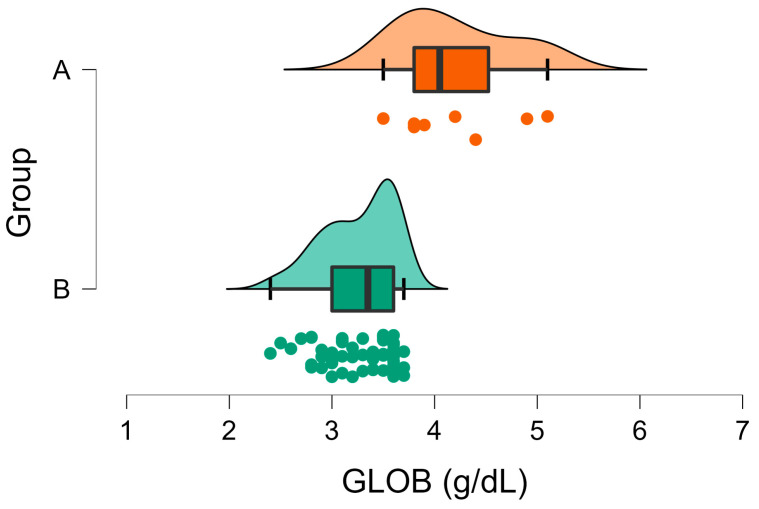
Raincloud plot of GLOB concentrations (U/l) in Group A (wild rabbits with positive *Eimeria* liver imprints) and Group B (wild rabbits with negative *Eimeria* liver imprints). The data distribution (cloud) with jittered raw data (the rain) and the central tendency and error (boxplot) are illustrated.

The GLOB concentrations were above the upper reference limit in all cases in Group A and in the majority (88%) of cases in Group B (Figure 7). The mean globulin concentration was significantly higher in Group A compared to Group B (Table 1, Figure 7).

**Figure 8:**
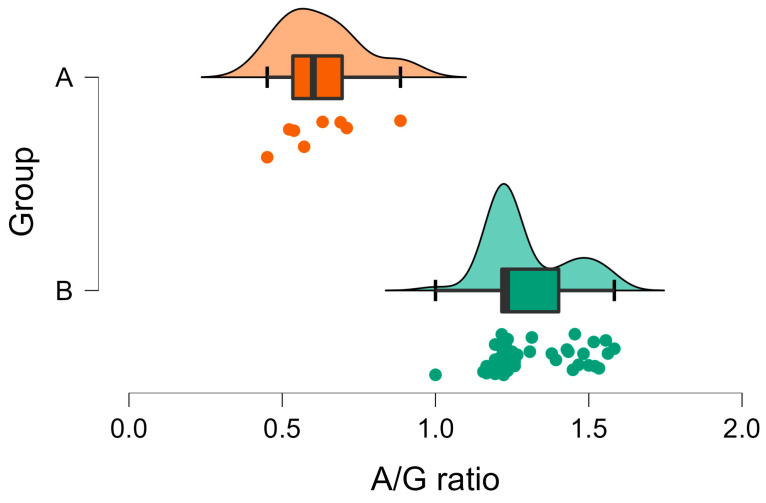
Raincloud plot of A/G ratio in Group A (wild rabbits with positive *Eimeria* liver imprints) and Group B (wild rabbits with negative *Eimeria* liver imprints). The data distribution (cloud) with jittered raw data (the rain) and the central tendency and error (boxplot) are illustrated.

The A/G ratio was below one in all Group A wild rabbits and above one in all Group B individuals (Figure 8). Moreover, a significant difference was observed between the mean A/G ratios in the two groups (Table 1, Figure 8).

## 4. Discussion

In this study, we showed that wild rabbits on the island of Lemnos, Greece, are naturally infected with *E. stiedae*. We also defined the biochemical profile of the infected individuals based on the analysis of specific variables that reflect liver injury and/or function.

Overall, 13.3% (8/60) of the liver imprints examined were positive for the presence of coccidial oocysts. Although not identified at a species level using a specific diagnostic tool, based on the current knowledge, we expect that they were *E. stiedae* oocysts. In addition, the different rabbit intestinal coccidian species parasitize distinct parts of the intestine and different parts of the mucosa, while *E. stiedae* is the only *Eimeria* sp that localizes the epithelium of biliary ducts in the liver [2].

Our results are in agreement with the limited number of studies that have been conducted in wild rabbit populations to date. In the UK, *E. stiedae* was detected in 24% of examined wild rabbits [9]. In Australia, oocysts of ten different *Eimeria* spp. were identified in wild rabbit feces with prevalence ranging from 7.4% for *E. coecicola* to 84.2% for *E. perforans* while the prevalence for *E. stiedae* was 44.7% [31]. In a 2003 study in France, researchers identified ten *Eimeria* spp. in wild rabbits, with prevalence ranging from 0 to 100% for different species and locations; the prevalence of *E. stiedae* ranged from 4 to 21% in adult individuals [32]. Another study showed that the prevalence of coccidial oocyst excretion in wild rabbits in Scotland was 73.7% [33]. Researchers in Iran found that, 31% of wild rabbits examined were positive for *Eimeria* spp. by floatation technique and six *Eimeria* species, including *E. perforans* (18.3%), *E. magna* (16.9%), *E. media* (14.1), *E. irresidua* (11.2%), *E. flavescens* (4.2%), and *E. coecicola* (2.8%) were identified [23].

A larger number of studies reported the detection of *Eimeria* spp. in pet rabbits worldwide. The detection rates were constantly high in the different studies, i.e., 41.9–56.4% in China [34,35], 65% in Japan [36], 100% of examined farms in Poland [37], 21.2% in Germany [38], 28–67% in Iran [11,39], 70.3% in Indonesia [40], 47.6% in Algeria [41] and 52.7% in Turkey [42]. Recently Sioutas et al. described an outbreak of deaths in one of the largest industrial rabbit farms in Ioannina, western Greece, which was caused by mixing infections with *Eimeria* spp., *Passalurus ambiguus* and *Cyniclomyces guttulatus* [43]. Previous studies in Greece have reported a high frequency of *Eimeria* spp. detection in other animal species: poultry (85.7%) [44], small ruminants (69%) [45] and fish (67.3%) [46].

In the majority of the above-mentioned studies in domestic and wild rabbits or other animal species, the diagnostic tools used were fecal examination with flotation techniques and/or molecular methods. In this study, we used liver impression smears which were Hemacolor stained, for the detection of *Eimeria* oocysts in the liver of wild rabbits; this technique is easy, time-saving, and it does not require specific equipment and trained personnel. Researchers in previous studies have successfully used the impression smear method for the diagnosis of hepatic coccidiosis following post-mortem examination and showed its utility and reliability for the detection of *E. stiedae* oocysts [3,22].

We also defined the serum biochemical profile of naturally infected wild rabbits based on the analysis of variables that reflected liver damage and dysfunction; the liver enzyme activities, that is, ALT, AST and GGT, were increased while the concentrations of ALB, GLOB and TP were decreased in the infected individuals compared to the non-infected ones. Statistically significant differences were found for all the above-mentioned biochemical variables between the infected and non-infected animals. Our findings are well conformed to the observations of previous experimental studies in domestic rabbits [4,8,14,15] and are suggestive of the effects of *E. stiedae* infection on liver function. For instance, the experimental study of Jing et al. showed that ALT, AST and GGT activities increased significantly and were indicative of the liver injury caused by *E. stiedae* infection and the parasite reproduction in liver parenchymal cells. In particular, the AST levels were seven-fold compared to the non-infected individuals [4]. In our study, the mean activities of ALT, AST and GGT in infected wild rabbits were higher compared to the non-infected ones, but they did not increase multifold. This finding should be interpreted with caution because we used reference intervals for domestic and alive rabbits. In a study in humans, statistically significant differences were observed between antemortem and postmortem ALT, AST and GGT liver enzyme activities [47]. However, the increase in enzyme activity after death seems to be time-dependent and contrary to the above-mentioned studies in humans; in our study, sampling was performed within 3 h after the rabbits were shot. Moreover, a comparison between the groups sampled after death negated the possible effect of postmortem sampling and, therefore, seemed to be more reliable. As for the liver enzyme activities in experimental studies, compared to the present study, the very short half-life of around five hours of both ALT and AST [48] and the unknown stage of infection in the present study could account for differences in the magnitude of the increase in the enzyme activities in infected animals.

The increased activity of ALT, an enzyme that is primarily found in the cytosol of hepatocytes, is an important biomarker for liver damage due to hepatocyte injury and inflammation in humans, dogs and cats. In rabbits, though, similar to other herbivores, ALT activity is of lesser value due to little tissue specificity and a shorter half-life. However, a correlation between the degree of liver necrosis to serum ALT activity has been shown for rabbits [49]. Raised ALT levels, bilirubin and GGT can be associated with hepatic coccidiosis [48]. Based on our results, the mean value for ALT was significantly higher in the infected wild rabbits. However, in the majority of infected wild rabbits, ALT levels remained within the reference intervals for domestic rabbits.

Regarding AST activity, this enzyme was widely distributed in many tissues in rabbits, including the cardiac tissue and muscle, as well as the liver—and it has a short half-life as well. Similarly to ALT, elevated AST levels may be found in rabbits diagnosed with liver damage [48,49]. In this study, the mean value for AST in infected rabbits was significantly elevated compared to the non-infected individuals (Table 1). Based on the depicted distribution of AST activities in both groups (Figure 3) and their differences, AST seemed to be a more sensitive marker of liver injury in infected rabbits. Similarly, it has been shown previously that feline peritonitis virus-infected cats developed pyogranulomatous hepatitis, which was characterized by mildly increased AST while ALT activity was normal [50].

The activity of GGT is generally low in rabbits. The elevated GGT activities in this species are associated with obstructive lesions of the bile ducts, and it is considered an important enzyme with which to estimate liver disease [48]. Similarly to the above-mentioned liver enzymes, the infected wild rabbits presented a higher mean GGT activity compared to non-infected individuals (Table 1), and the GGT levels were outside their reference intervals in 100% of the infected wild rabbits.

TP and albumin concentrations were decreased in infected individuals, while the globulin concentrations were increased (Table 1). The observed hypoproteinemia is the result of the reduced albumin fraction of proteins. In addition, the liver is the only site of albumin synthesis and decreased ALB concentrations in rabbits that may be associated with advanced hepatic diseases, such as hepatic coccidiosis or scarring and necrosis [43]. Moreover, decreased ALB concentration in infected wild rabbits can be attributed to the downregulation of its transcription due to the release of cytokines from macrophages as part of the acute phase response caused by the parasitized liver inflammation and damage. The acute phase reactant response may also lead to the increased degradation of albumin [51]. On the contrary, the acute phase reaction was accompanied by an increase in certain globulin fractions, while the combination of low albumin and high globulin concentrations resulted in a decreased A/G ratio. Similar results of ALB and GLOB concentrations have been reported in rabbits experimentally infected by *E. stiedae,* along with their combined effect on the A/G ratio [52]. Regarding postmortem sampling, it has been reported that the postmortem total protein and albumin concentrations are reliable surrogates for ante mortem corresponding values in humans independent of the time of postmortem sampling [47].

Our findings clearly support that, in terms of the comparison between infected and non-infected wild rabbits, the liver enzymes activities, that is, ALT, AST and GGT, were increased while the concentrations of ALB, GLOB and TP were decreased in the infected individuals compared to the non-infected ones. However, the clinical interpretation of biochemical parameters based on the reference intervals for domestic rabbits was quite challenging and inconclusive for hepatic coccidiosis in wild rabbits. This observation could be attributed to the validity of the reference intervals. Foremost, the reference intervals used in this study were for domestic rabbits because no reference interval studies have been conducted up to now for wild rabbits. In addition, by definition, the reference intervals do not reflect the whole population, and it is expected that 5% of all results from “healthy” individuals fall outside the reference intervals [53]. Moreover, normal physiological processes, genetic differences, environmental factors and pathology are some of the reasons that could potentially lead to variations between and within the subjects involved in a reference interval study and consequently affect the validity of the reference intervals [54]. Additionally, despite the fact that postmortem sampling seemed not to affect liver enzymes, the possibility of some variation in the reference intervals cannot be excluded [47]. Finally, this study was conducted in a natural population of wild rabbits, and other reasons affecting liver function cannot be ruled out.

Except for hepatic coccidiosis, elevations in liver enzymes and hypoproteinemia are expected in cases of hepatic damage, necrosis, biliary obstruction and cholestasis [55]. When liver disease is suspected in domestic rabbits, the differential diagnostic approach includes clinical examination and a broad range of diagnostic tests that are available in clinical practice [55]. In the case of studies conducted in wild populations, the available diagnostic tools are limited, and the value of necropsy, cytology and basic blood work is greatly highlighted. With that in mind, and taking into account that no evidence of any other disease was detected macroscopically during liver sampling, we cannot definitely exclude the sum of the potential causes of liver disease in this wild population, especially those that are microscopically detected; however, we used a simple, easy and quick technique to detect *E. stiedae* infection which stood at the top of a prioritized differential list.

In Greece, the geographical distribution of the wild rabbit population is limited to the island ecosystems, while this species is absent from the mainland. Over the last two decades, the wild rabbit has overpopulated Lemnos Island, causing devastating effects on the rural economy and is considered a threat to biodiversity, ecosystems, and crops on the island [56,57]. Consequently, the national legislation of Greece has made provisions for the legal hunting of wild rabbits from late August to early March in this specific area (FEK 3515 B’ 2 August 2021), aiming to control overpopulation via hunting pressure. This wild rabbit population gained scientific attention in terms of their geographical distribution and the reasons that led to their overpopulation, its effects on agro-ecosystems [56,58], the body condition variation in the population, and the possible management plans to ensure control or conservation [59], including the way that this problem could be turned to opportunity through marketing and tourism [60] and its veterinary and public health importance [61,62].

To the best of our knowledge, only two studies have been conducted up to this point to investigate the exposure of this population to important pathogens. The 2019 study by Tsakmakidis et al. showed that 1% of the wild rabbits sampled during two hunting seasons were exposed to *L. infantum* [62]. Later, Athanasiou et al. reported that 4.2%, 5.5%, 18% and 9.7% of the wild rabbits included in the study were seropositive to *L. infantum*, *T. gondii*, *A. phagocytophilum* and *B. caballi*, respectively [61]. This study adds to the current knowledge of the pathogens affecting this species and those circulating in this wild rabbit population on the island of Lemnos, Greece. Moreover, it suggests that *E. stiedae* infection exerts pathological effects on the liver function of wild rabbits, which are reflected in the liver variables.

## Figures and Tables

**Table 1:** Mean values and standard error for the biochemical variables examined (ALT, AST, GGT, TP, ALB, GLOB and A/G ratio) in the infected (Group A) and non-infected (Group B) groups of wild rabbits and reference intervals for domestic rabbits (*O. cuniculus*).

Biochemical Variable	Group	Mean	SE	*p*-Value	RI
ALT (U/L)	A	32.90	1.11	0.005	31–53
	B	43.63	6.22		
AST (U/L)	A	**26.94**	1.06	<001	42–98
	B	**104.13**	12.12		
GGT (U/L)	A	**10.50**	0.43	<001	0–7
	B	**17.50**	3.39		
TP (g/dL)	A	**7.44**	0.08	0.003	5.5–7.2
	B	6.78	0.20		
ALB (g/dL)	A	4.19	0.04	<001	2.7–4.6
	B	**2.58**	0.12		
GLOB (g/dL)	A	**3.26**	0.05	<001	1.5–2.8
	B	**4.20**	0.20		
A/G ratio	A	1.30	0.02	<001	N.A.
	B	0.63	0.05		

SE = standard error, RI = reference interval, ALT = alanine aminotransferase, AST = aspartate aminotransferase, GGT = gamma glutamyl transpeptidase, TP = total protein, ALB = albumin, GLOB = globulin, A/G ratio = albumin to globulin ratio N.A. = not available. Values in bold = mean values outside the reference intervals.

## Data Availability

The data presented in this study are available on request from the corresponding author. The data are not publicly available due to further processing for other studies.
